# Barriers and enablers to sexual health service use among university students: a qualitative descriptive study using the Theoretical Domains Framework and COM-B model

**DOI:** 10.1186/s12913-018-3379-0

**Published:** 2018-07-24

**Authors:** Christine Cassidy, Andrea Bishop, Audrey Steenbeek, Donald Langille, Ruth Martin-Misener, Janet Curran

**Affiliations:** 1 School of Nursing, Dalhousie University, 5869 University Avenue, PO BOX 15000, Halifax, NS B3H 4R2 Canada; 2 IWK Health Centre, Halifax, NS Canada; 3 Department of Community Health and Epidemiology, Dalhousie University, Halifax, NS Canada

**Keywords:** Sexual health services, Sexually transmitted infections, Reproductive health, University students, Theoretical domains framework, behaviour change wheel, Qualitative research

## Abstract

**Background:**

University students are within the age group at highest risk for acquiring sexually transmitted infections and other negative health outcomes. Despite the availability of sexual health services at university health centres to promote sexual health, many students delay or avoid seeking care. This study aimed to identify the perceived barriers and enablers to sexual health service use among university undergraduate students.

**Methods:**

We used a qualitative descriptive design to conduct semi-structured focus groups and key informant interviews with university students, health care providers, and university administrators at two university health centres in Nova Scotia, Canada. The semi-structured focus group and interview guides were developed using the Theoretical Domains Framework and COM-B Model. Data were analyzed using a directed content analysis approach, followed by inductive thematic analysis.

**Results:**

We conducted 6 focus groups with a total of 56 undergraduate students (aged 18–25) and 7 key informant interviews with clinicians and administrators. We identified 10 barriers and enablers to sexual health service use, under 7 TDF domains: knowledge; memory, attention and decision-making processes; social influences; environmental context and resources; beliefs about consequences; optimism; and emotion. Key linkages between students’ social opportunity and motivation were found to influence students’ access of sexual health services.

**Conclusions:**

We identified barriers and enablers related to students’ capability, opportunity and motivation that influence sexual health service use. We will use these findings to design an intervention that targets the identified barriers and enablers to improve students’ use of sexual health services, and ultimately, their overall health and well-being.

**Electronic supplementary material:**

The online version of this article (10.1186/s12913-018-3379-0) contains supplementary material, which is available to authorized users.

## Background

The transition from adolescence to adulthood is a complex and exciting time for young adults as they begin to explore their sexual identity and sexual relationships [1]. Although healthy sexual relationships yield many physical and emotional benefits [2, 3], young adults are at risk for acquiring sexually transmitted infections (STIs) and other negative sexual health outcomes [4]. Evidence indicates that young adults aged 20 to 24 are more susceptible to contracting STIs than any other age group [4–6]. These outcomes are of significant concern: If left untreated, STIs can lead to serious health consequences, especially for women, including pelvic inflammatory disease, ectopic pregnancy, and infertility [4].

University students may be at an increased risk of acquiring STIs due to pressure to engage in high-risk behaviours, including excessive alcohol consumption [7], casual sex, and inconsistent condom use [8]. As such, many university and college campuses offer a range of sexual health services to prevent and treat STIs, decrease the risk of the health consequences of STIs, and promote positive sexual health practices among students [9]. Sexual health services include: health education, student outreach, STI testing and treatment, peer education, and condom distribution [10]. University health services are viewed as ideal ‘health care homes’ for students living away from their usual primary care providers [10]. Despite the existence of such services, many university students often delay or avoid seeking sexual health services. In the United States, only 27% of university students report having ever accessed sexual health services [11]. In Nova Scotia, Canada, only 22% of female undergraduate students and 8% of male undergraduate students report having ever accessed their university’s sexual health services [12].

Multi-level barriers and enablers are known to influence sexual health service use among university students and provide some insight into the low rates of service use. Studies have predominantly focused on individual and interpersonal-level factors from the perspective of post-secondary students and young adults, including biological sex, ethnicity, age, perceived risk, stigma, and perceived norms [13, 14]. Perceived barriers to sexual health services among young adults include: service access (i.e., location, hours, confidentiality), service entry (i.e., waiting time, waiting environment, fear of being seen), quality of services (i.e., health care provider characteristics) and personal factors (i.e., stress associated with seeking sexual health services) [15]. Few studies have explored health service-level factors from the perspective of health care providers, administrators or policy-makers [16, 17]. Further, there is a paucity of literature on how university students’ developmental stage, the university context, and health service characteristics merge to create barriers and/or enablers to university students’ use of sexual health services. Research efforts are needed to understand the barriers to service to in order to decrease the risk of the health consequences of STIs and promote positive sexual health practices among students.

Many researchers and organizations, including the Medical Research Council [18] and the National Institute for Health and Care Excellence (NICE) [19] in the United Kingdom, propose that interventions are more likely to be effective if theoretical models are used in intervention development. The Behaviour Change Wheel (BCW) is one such approach [20]. It is a systematic, theory-based guide to intervention design based on the principles of the COM-B model, which suggests that for any behaviour to occur there must be a change in one or more of the following: capability, opportunity or motivation. The COM-B model has also been used alongside the Theoretical Domains Framework (TDF) to better understand the influences on the target behaviour [20]. The TDF is a behavioural framework consisting of 14 domains that expands on the COM-B components and captures potential mediators of behaviour change [21]. While the BCW has been used to design interventions in many contexts, such as smoking cessation [22], alcohol reduction [23], condom use [24], and sexual counselling [25], it has yet to be applied to the use of sexual health services by university students. Therefore, the aim of this study was to use the COM-B model and TDF to identify barriers and enablers for students’ use of sexual health services on campus to inform the design of future interventions to promote sexual health service use among university students.

## Methods

### Design

A larger, three-phased mixed methods study is being conducted to develop a theory-based intervention to improve university students’ use of sexual health services. Full study methods and Phase 1 study results have been published elsewhere [12, 26]. This present study was Phase 2 in the intervention design process. We used a qualitative descriptive design [27, 28] to conduct semi-structured focus groups with students and key informant interviews with health care providers and administrators to identify barriers and enablers to sexual health service use among university undergraduate students.

### Setting

Participants were identified from two universities in Nova Scotia, Canada that offer on-campus sexual health services [26]. University A is large urban university, with approximately 13,600 undergraduate students (45% male; 55% female). University A’s health centre staffs nine full-time physicians, three registered nurses, and one advanced practice nurse. University B is a small, rural university, with approximately 3500 undergraduate students (42% male; 58% female) [29]. University B’s health centre staffs five part-time physicians and one full-time registered nurse. Both universities offer general health care and sexual health services to their student populations, including STI/HIV and Pap testing, sexual health education, birth control counselling, and emergency contraception.

### Participants

Focus group participants were university undergraduate students, aged 18–25, from the two universities who had or had not accessed their university sexual health services in the past. In our Phase 1 analyses, we found different patterns of sexual health service use among students who self-identified as male, female, and members of the LGBTQ community [12]. As a result, we used a stratified purposive sampling strategy with snowballing sampling techniques [30] to identify participants from these three subgroups. Recruitment posters were posted across University A and B campuses, including libraries and student union buildings. An email describing the study and invitation to participate was distributed to various student organizations (e.g., student union, LGBTQ student organizations). Interested participants contacted the research assistant (RA) via email. The RA responded with study information and a screening questionnaire to determine eligibility (age, year of study, preference for male, female, or LGBTQ focus group). Once eligibility was confirmed, the RA sent the date and time of the focus group and consent form to the participant. Interview participants were health care providers (physicians and nurses) and administrators (directors and managers) from the two university health centres. An email invitation was sent to the clinic managers and distributed to potential interview participants. Interested participants contacted the RA via email to set up an interview time.

### Procedure

Focus group and interview guides were developed based on the COM-B model of behaviour and the 14 domains included in the TDF (Additional file 1). We developed two to three questions per domain using existing guidance from Atkins et al. [31] Additional prompts were prepared to probe domains if further clarification was needed. Further, we added questions within the TDF domains that probed or expanded on Phase 1 results [12]. We tested the focus group guide with three university undergraduate students and interview guide with one administrator to identify any confusing terminology or concerns about the questions. The focus group and interview guides were then refined based on the feedback.

#### Student focus groups

A consent form was reviewed and signed by each participant prior to each focus group. Students were provided an honorarium for their participation in the form of a $30 grocery store gift card. The focus groups lasted between 40 and 60 min. All focus groups were audio-recorded, transcribed verbatim and anonymized prior to analysis. Additional field notes were also taken by either the RA or interviewer.

#### Health care provider and administrator interviews

The consent form was sent via email to participants prior to the interview. It was reviewed at the beginning of the interview and verbal consent to participate was obtained. Health care provider and administrator participants were offered a $10 honorarium for their participation. The interviews lasted between 15 and 30 min. They were audio-recorded, transcribed verbatim, and anonymized prior to analysis.

### Data analysis

Focus group and interview transcripts were combined to provide one complete dataset for analysis. Data were analyzed using a directed content analysis approach [32] followed by inductive thematic analysis [31, 33]. All transcripts were coded in NVivo 11 [34]. First, two reviewers (CC, AB) read the transcripts and categorized similar statements into the three COM-B categories and further into the 14 TDF domains. One reviewer (CC) coded all focus groups and key informant interviews, while a second reviewer (AB) independently reviewed three focus group transcripts and two interview transcripts. Coding stripes on NVivo were compared for consistency in coding and a codebook was finalized for the remaining analyses. Second, an inductive coding approach was used to generate subcategories of participants’ specific beliefs within the initial coding scheme of the 14 TDF domains. A specific belief is a group of similar responses that suggest the belief may influence the target behaviour [31]. Third, the coded data were further inductively examined to generate themes that represent the barriers and enablers perceived to influence students’ sexual health service use. Lastly, the student focus group and health care provider/administrator key informant interview data were compared for areas of agreement, partial agreement, silence, or dissonance between findings from the students focus groups and health care provider/administrator key informant interviews [35].

### Member checking

Following the deductive and inductive data analyses, we brought the initial themes to a group of students at each university for member checking. Member checking involves verification of the emerging themes and inferences, and provides participants with the opportunity to offer clarification, add information, and prioritize the initial themes [36, 37].

## Results

We conducted six focus groups, including one with male students, one with female students, and one with student members of the LGBTQ community, from each of the two universities (*N* = 56). Further, we conducted seven key informant interviews with two administrators, three physicians and two nurses (Table 1).

**Table 1:** Focus group and key informant interview participants

Participants	University A	University B
Focus Groups (*N* = 56)		
Male Students	10	9
Female Students	14	12
LGBTQ Students	6	5
Total	30	26
Key Informant Interviews (*N* = 7)		
Administrators	1	1
Physicians	3	0
Nurses	1	1
Total	5	2

Following data analysis, we conducted two member checking exercises with a group of seven University A students and four University B students. These students had also participated in the original focus groups. All students confirmed that our understanding of their perceived barriers and enablers to sexual health service use were accurately reflected in these initial themes. Together, minor refinements were made to the wording of the themes to better reflect their perspectives on sexual health services and students further described relationships between the themes. Overall, the focus group and interview participants identified several barriers and enablers to university students’ use of sexual health services. Below we describe how the data align with the COM-B model and TDF (Tables 2 and 3).

**Table 2:** Barriers and enablers to sexual health service use among university undergraduate student

COM-B	TDF Domain	Themes	Belief Statements	Participant^a^	
				Students	HCP/Admin
Capability	Knowledge	1. Limited sexual health knowledge and awareness	Knowledge and awareness of the services is important to know when and how to access First year students lack sexual health-related knowledge and find it difficult to remember where to go or how to access services Students have questions but do not know where to go, which can lead to a cycle of misinformation Students have go-to informants for sexual health information, including Residence Assistants (RAs) and the internet	✔	✔
		2. Lack of clarity for LGBTQ students	LGBTQ students do not always understand what they are at risk for or what services they should be accessing Some health care providers do not feel confident providing sexual health care to LGBTQ students	✔	✔
	Memory, Attention, Decision-Making Processes	3. Visibility of sexual health services	Certain prompts and reminders help students to remember to access their sexual health services, including emails, posters, Facebook groups Sexual health service use can be a game of hide and seek – students have to go searching for information related to the health clinic	✔	✔
Opportunity	Social Influences	4. Health care provider interaction	Students favour seeing the same health care provider for continuity in their care Student-HCP interaction (both positive and negative) during a sexual health visit impacts their experience with care and willingness to return	✔	✔
		5. Peer influence	Supportive friends promote access of sexual health services There is a stigma related to accessing sexual health services which prevents service use Seeing classmates at the clinic is uncomfortable Female students felt a sense of responsibility to access sexual health services to protect both themselves and their partner’s health.	✔	–
	Environmental Context and Resources	6. Campus culture	University culture promotes sexual experimentation and exploration, risk taking behaviour, and avoidance of health promotion behaviours such as sexual health service use It is important to have sexual health services available in an environment that promotes risk-taking behaviour	✔	–
		7. Accessibility of services	Financial access: students are paying into the wellness fund, so they feel as so they should use the services Hours of operation can help or hinder students’ access depending on their flexibility Location of services is an important characteristic Wait times hinder students’ access; students are forced to miss class due to wait times	✔	✔
Motivation	Beliefs about Consequences	8. Period of exploration and experimentation	University is a time of sexual exploration and risk-taking behaviours; it is important to have these services available during this period	✔	–
	Optimism	9. Normalizing sexual health	Some students are seeing trends towards normalizing sexual health and access of sexual health services There is a trend towards sex-positivity which supports service use	✔	✔
	Beliefs about Consequences and Emotions	10. Stigma, privacy and confidentiality	There is still a stigma related to accessing sexual health services Students feel a range of emotions when accessing sexual health services (awkward, discomfort, frustration, shame) Services that value privacy and confidentiality can mitigate the negative emotions	✔	✔

^a^✔ = Agreement by participants; − = Silence by participants; *HCP*, health care provider; *Admin,* university administrator

**Table 3:** Barriers and enablers to sexual health service use: Salient domains from the TDF mapped to the COM-B

Barriers and Enablers	COM-B and TDF Domains						
	Capability		Opportunity		Motivation		
	Psychological		Social	Physical	Reflective		Automatic
	K	MAD	SI	E	CO	OP	EM
Limited Sexual Health Knowledge	✔						
Lack of Clarity for LGBTQ Students	✔						
Visibility of Sexual Health Services		✔					
Health Care Provider Interaction			✔				
Peer Influence			✔				
Campus Culture				✔			
Accessibility of Services				✔			
Period of Exploration and Experimentation					✔		
Normalizing Sexual Health						✔	
Stigma, Privacy and Confidentiality					✔		✔

*Note. K* knowledge, *MAD* memory, attention, and decision-making processes, *SI* social influences, *E* environmental context and resources, *CO* beliefs about consequences, *OP* optimism, *EM* emotion

### Capability

Students’ psychological capabilities influenced their use and non-use of sexual health services on campus. Psychological capability is defined within the COM-B model as the capacity to engage in the necessary thought processes, such as comprehension and reasoning [20].

#### Limited sexual health knowledge and awareness

Student participants identified their lack of knowledge and awareness of sexual health services, particularly during their first year of undergraduate studies, as an important barrier. Students felt overloaded with new information during their first-year orientation and found it difficult to remember information related to sexual health services throughout the year. Participants also reflected on questions they had related to sexual health but did not know where to seek information, which often leads to a “cycle of misinformation”.

*“And a lot of students come from out of province, and they’re here, and they’re just like, ‘Wait, I have to go to the hospital to do this?’ And it becomes like a cycle of misinformation. And it took me a long time to figure out all those things.”* – University A FG #1

Students would often seek out key informants (e.g., residence assistants, peers) with their questions related to sexual health services. These key informants were deemed to be an important enabler of sexual health services.

*“I found that when I was a resident at least, and this was only a year ago, that the RAs [Residence Assistants] were great with making us aware of like consent and sexual health awareness and stuff like that…the RAs are primarily where I got the information about where to go and who to see*.” –University B FG #2.

Health care provider and administrator participants also stressed the need to enhance sexual health promotion and education amongst university students, particularly for students entering their first year.

#### Lack of clarity for LGBTQ students

Participants from the LGBTQ community described a lack of clarity regarding when and why they needed to access sexual health services. Students stated that they do not always understand what STIs they are at risk for contracting or transmitting. This is further complicated by their interactions with health care providers who are also not always clear on what LGBTQ students need with respect to STI testing.

*“I found there’s been like an interesting assumption that like I know what I need to be tested for. Like I’ve been asked like, ‘Oh, do you want to be tested for HIV, do you want…’ And I’m like, ‘I don't know what I need to be tested for.’ Especially because like as a woman who sleeps with women, it’s like I don’t really know. We don’t really have a lot of education around what we could be exposed to. So I’m just kind of like, ‘Test me for what you think I need to be tested for.’”* - University A FG #3

Some health care providers have specialized training in sexual health care provision for LGBTQ patients. Other health care provider participants described themselves as less confident with caring for LGBTQ students and sought out colleagues with advanced training in LGBTQ health to ask questions.

*“And sometimes for me, like I don’t have a lot of experience with like the trans community and those different types of communities. So sometimes I’m uncomfortable.” –* University B Health Care Provider Interview

#### Visibility of sexual health services

Students believed that enhanced visibility of sexual health service information would help to improve students’ access. Some students felt that they were playing a game of ‘hide and seek’ when trying to access sexual health services, as they had to go searching for information.

*“Like my partner and I have like actually searched for it, and we couldn’t find it. So we ended up just going to the doctor. But we’ve actually been looking for it and we just didn’t know where to check.” –* University A FG #1

Participants recommended using prompts and reminders to improve access and promote visibility. Students suggested regular emails and posters with sexual health service information and having recurring mobile clinics in high-traffic areas and at consistent times to promote visibility and accessibility of the services. Similarly, clinician and administrator participants also identified the need for improved advertisement. One administrator at University B stated: *“[We] need to highlight who we are, where we are, and what we do.”*

### Opportunity

Barriers and enablers within the social and physical university environment shaped the opportunities for students’ use of health services. Social opportunity refers to the social factors that influence the way that we think about things (i.e., cultural norms, social cues). Physical opportunity is afforded by the environment (i.e., time, location, resources) [20].

#### Health care provider interaction

Student participants recalled their previous experiences with the university health clinic and how it influenced their perceptions of sexual health services. Student-health care provider interactions (both positive and negative) during a sexual health-related visit impacts students’ experience with care and willingness to return. For example, students favoured seeing the same clinician at each visit because it provided them with an opportunity to build a trusting relationship.

*“I’ve had like situations where… well, like the doctor that I see regularly, he always is like if there's something wrong like I’ll call you. And I guess I have a relationship with him that way so I don’t mind waiting in that way.” –* University A FG#2

*“I had a bad experience with one particular doctor, and I didn’t know which days they would be working. And if I needed to go that day, and there were the only one working, then I wouldn't want to go there.” -* University B FG #1

LGBTQ students also identified their interactions with health care providers as an important barrier to accessing sexual health services. Students stated that their health care providers often assume they are in a heterosexual relationship, and subsequently, they are frustrated when they have to reiterate their sexual orientation at each visit. Participants stated these interactions added further confusion to their visit, and negatively influenced their willingness to return.

*“Even though I go to the same doctor, she often forgets that I’m gay. And so I repeatedly have to come out to her in terms of like if … Like she’ll just see my file and see that I’m not on birth control, and she’ll be like, “Why aren’t you on birth control?” … And I have to like disclose again. And it’s just kind of uncomfortable because it’s like why don’t you remember this?” -* University A FG #3

Findings from health care provider interviews also highlighted the importance of building trusting relationships between clinicians and students. Health care provider participants reiterated the importance of the nurse-student relationship, as they are often the first point of contact for students and have more time to spend with patients. They found that avoiding medical jargon and using a common language with students was useful for building relationships with students. Participants also stated that continuity of care is critical to encourage students, particularly LGBTQ patients, to return to the clinic.

*“I think being able to talk to a student in a language…. to be able to find a common language. Because you know, if you’re just using very medical terminology, that doesn’t always…it’s not always understood by the patient.” –* University A Health Care Provider Interview.

#### Peer influence

Students identified the positive and negative influence of peers on their use of sexual health services. Several students described accessing sexual health services as a social activity, where they support one another by going to the clinic together.

*“Any time that I know that there’s a pop-up clinic or anything going on, like I’ll text my roommates and be like what’s happening. I mean it’s not related to that but like it’s just… it’s kind of a fun thing to do together…. and you know, you can make a little date out of it with friends.” –* University A FG#1.

Other students described the stigma related to accessing sexual health services, specifically focusing on the discomfort of seeing other classmates at the clinic. This barrier was especially relevant for participants from University B, where knowing other students on campus was highly probable and accessing sexual health services could impact their social status or how they were viewed amongst their peers. For example, one male student at University A stated: *“You don’t want to be that guy…. that guy with an STD. Nobody wants to be patient zero.”* Another student described the stigma from their perspective:

*“I mean there's still a stigma around people going to access these services and just people as sexual human beings. So I think when you have it on campus, there's always a fear that you're going to bump into someone that you know, and you don't know how they’re going to receive that. I think most of the people are like, “Good for you.” Like that’s a good thing to go do. But you never really know how people are going to react and who you’re going to see there.” –* University A FG #3

#### Campus culture

Students expanded on the influence of peers and described that the campus culture promotes partying and risk-taking behaviours, such as alcohol and drug use, casual sex, and inconsistent condom use. Students believed this environment does not always support health promotion behaviours and can lead to the avoidance of sexual health services. Students highlighted the importance of having a safe environment, such as accessible sexual health services, to engage in risk-taking behaviour.

*“And I agree, like I think it’s super important at this stage especially just because like of different things that come with the culture and experimenting.” –* University A FG #2

*“Like obviously if you’re like sexually active and like you're engaging in multiple partners, like because this is university and everyone’s so out there and experimenting with so many different things, that like it’s good to go get yourself checked out and like make sure your partners are checked out.” –* University B FG #3

#### Accessibility of services

The accessibility of sexual health services was seen as both a barrier and enabler to students’ use. Services are financially accessible, as students do not have to pay out-of-pocket for services. Further, some participants felt compelled to use the services since they were paying into a wellness fund each semester. The location of services was seen as an important aspect for many students. University A students valued having a clinic that was visible on campus and was seen as a safe and welcoming place.

*“At the same time, I like accessing services on campus because I feel like campus is a safe place. Like I’m here every day and I love it, I’m familiar with it. So I like it in that sense.”* – University B FG #1

University B students, however, felt that they had to go searching for the clinic as it was not clearly visible on campus. This in turn created an unwelcoming atmosphere.

*“It’s right underneath [Building Name]. So like it’s right underneath like a res[idence]. And it’s like it’s just an awkward placement. And it’s not really like there it is. Like you have to like really walk by and then see it.” –* University B FG #2

Clinic hours of operation can also help or hinder students’ access of sexual health services. Student participants described difficulties with accessing services that are only open during class times. University A students appreciated the opportunity to schedule appointments in the evenings and on weekends. This service was not available to University B students who were then faced with having to decide whether to miss class in order to access the services. Similarly, students discussed how they are often forced to miss class due to wait times, which in turn, impacted the likelihood of them returning to the clinic.

*“The only time I went to the on campus health clinic for sexual health, I waited there for probably about an hour and a half or 2 h. And I was missing my classes. And I went up to the receptionist and I said, you know, I’m missing my classes. You know, I have a quiz today. I can’t just, you know, skip my quiz but I need this [STI] test. And she said, “Oh, like I can try but I can’t do anything for you.” So I left and I never went back there. Because like when can you find the time to again skip your classes” –* University B FG #1

Health care provider and administrator participants recognized that hours of operation make it difficult for students to access the clinics. To improve accessibility, both universities employ registered nurses to provide student outreach and sexual health promotion and prevention initiatives across campus. As well, providers at University A indicated the presence of weekly mobile STI testing clinics helped to facilitate students’ access of sexual health services.

### Motivation

Several barriers and enablers tapped into students’ motivations, which are defined as the brain processes which direct our decisions and behaviours. The COM-B model differentiates between automatic motivation (i.e., emotions and impulses) and reflective motivation (i.e., evaluations and plans) [20].

#### Period of exploration and experimentation

Student participants described their university experience as a period of sexual exploration and experimentation, which was seen as a motivator for accessing sexual health services. Since sexual experimentation and exploration is a normal aspect of growth and development, students believed it to be important to have sexual health services available to them during this time.

*“It’s needed, point blank. Especially I think at this age where, I don't know, people I guess are maybe experimenting.… And like trying different things like meeting people and all that kind of stuff. So it puts you in situations where you need those kind of services maybe more so than at other stages in your life.” –* University A FG #1

#### Normalizing sexual health matters

Participants described the importance of normalizing sexual health matters to improve access to sexual health services. Students are starting to see trends towards normalizing sexual health and creating a sex-positive environment. Further, while female students in heterosexual relationships indicated they felt that the responsibility for STI testing currently lies with them in their relationships, they were optimistic that with enhanced sex positivity there may be a shift toward a shared responsibility amongst male partners.

*“I think I’m optimistic just because of how normalized it is around campus. And I think like the pop-up clinics do a really good job of normalizing it. And like I know res[idence] life and having those like let’s talk about sex things, it really opens the conversation.” –* University A FG #1

#### Stigma, privacy and confidentiality

Student participants described the stigma related to accessing sexual health services which can lead to a range of emotions including discomfort, frustration, and shame. A lack of privacy and confidentiality when accessing the services can jeopardize students’ satisfaction with care and willingness to return and leads to these negative emotions. When students feel their privacy and confidentiality is maintained, they are more comfortable with accessing the services.

*“I don’t like seeing other students, especially if I’m there for sexual health reasons. And I’ve had bad experiences in the past where they would say out loud that like I’m there for a pap test. And it's a small place. So like people in the waiting room could hear that. And it just made me uncomfortable.” –* University B FG #2

Health care provider and administrator participants also recognized the importance of maintaining privacy and confidentiality with university students. They identified this as a critical component to building a trusting relationship.

*“Because there’s an awful lot of personal anxiety around sexual health. Clearly there are barriers to conversation and communication. So obviously stressing confidentiality and expressing some comfort in conversation is important for them to open up about their own anxiety and concern.” –* University B Health Care Provider Interview

## Discussion

In this study, we used the COM-B model and TDF to identify barriers and enablers to sexual health service use from the student, health care provider, and administrator perspective. Our findings illustrate barriers and enablers at the individual, interpersonal, and health service levels. The COM-B model and TDF enabled a comprehensive theoretical analysis of university students’ capability, opportunity, and motivation and how these components work together to influence their sexual health behaviours.

Our findings suggest that limited sexual health knowledge is a barrier to sexual health service use among university students. Carroll and colleagues [38] found similar results in a systematic review of the reasons for use and non-use of school sexual health services among young adults: participants did not use the services because they were unaware that services existed or did not know what was available. As our study participants identified, students enter into their first year of university with diverse sexual experiences and varying levels of sexual health knowledge. Many participants were not aware of the sexual health services that are provided on campus or the reasons for accessing these services when they started their university journey. These findings expand on our previous quantitative results where undergraduate students in higher years of study were more likely to access sexual health services on campus [12]. To date, passive advertisement strategies have been used to target students in early years, including posting sexual health service information online and the inclusion of a health services pamphlet in students’ orientation packages. One way to improve students’ capability of accessing sexual health service use is to provide more targeted education initiatives with respect to availability of health care services and how to access these services. For example, student participants recommended delivering prompts or reminders of key messages throughout the year to avoid being overwhelmed with new information during their first week of orientation.

Previous research has found that non-heterosexual young adults and university students are less likely to access sexual health services [39, 40]. We found similar results in our quantitative study where non-heterosexual female students were 63% less likely to access sexual health services on campus compared to heterosexual students, and non-heterosexual male students were 79% less likely to access sexual health services on campus compared to heterosexual male students) [12]. LGBTQ participants in the current study were uncertain about when to access sexual health services and did not know what illnesses they were at risk for. Further, our results support previous research on health care providers’ perceived challenges with providing LGBTQ health care [41–43]. These findings suggest that addressing both student and health care providers’ capabilities, including knowledge on LGBTQ health, and promoting a welcoming, nonjudgmental, and confidential environment may facilitate students’ sexual health service use.

Students also described the physical opportunity, including service accessibility and campus culture, as both a barrier and enabler to sexual health service use. Because the campus culture promotes risky behaviours and avoidance of health promotion behaviour, student participants described the importance of having accessible sexual health services, including flexible hours of operation, convenient location, and mobile clinics. Service access is well-documented in the literature as a common barrier and enabler of sexual health service use among young adults and university students [11, 38]. Our findings suggest that service providers need to ensure sexual health services are delivered in a safe, accessible environment before they can tap into students’ motivations for accessing the services.

The findings indicate a strong link between students’ social opportunity and their motivation to access sexual health services. Student participants placed both positive and negative peer influence at the core of the relationship between social opportunity and motivation. Evidence has shown that peer norms influence university students’ attitudes and behaviours as they navigate the emerging adulthood developmental stage and begin to address issues of identity and intimacy [44–46]. This helps to explain the value our participants placed on privacy and confidentiality of the services to avoid being seen by their peers. This is a consistent finding in the sexual health literature, particularly with young adults [38] and university students [47, 48]: A lack of privacy and confidentiality can lead to feeling stigmatized, uncomfortable, judged, and shameful and an unwillingness to access sexual health services [46, 49]. Student participants also indicated that peer support helped to normalize sexual health. Students felt comfortable discussing sexual health matters with their peers and accessing health services together. Similarly, studies have found that social support can influence help-seeking attitudes and behaviours [46, 50, 51] and the likelihood of being tested for STIs [52].

Health care provider-student interaction was also seen as both a barrier and enabler to sexual health service use. Student participants described their relationship with their health care provider as an important factor in deciding whether to return to the clinic. Our findings are supported by a previous systematic review of young peoples’ views on the reasons for use and non-use of school sexual health services [38]. The review found that participants accessed sexual health services because the staff were welcoming, comforting, friendly, nonjudgmental, and good listeners. Similarly, findings from the World Health Organization show that young people report staff attitudes as the most important issue that attracted them to the health service or that led them to return [53]. Overall, social opportunity for students to access sexual health services appears to exist as a spectrum with stigma on one end and supportive relationships on the other. Future interventions should aim to overcome the social barriers and leverage the social enablers to motivate students to access sexual health services.

### Limitations

Study findings must be interpreted with the following limitations in mind. First, we recruited participants from two universities in Nova Scotia, Canada, which may not be representative of universities in other provinces and countries. However, through our inclusion of both a rural and urban university, the transferability of our findings may be improved. Second, our focus group methods may have introduced social desirability bias. We aimed to mitigate such bias by conducting separate focus groups for different subgroups. Third, due to challenges recruiting part-time clinicians from a small population (*N* = 6), only one clinician participated from University B.

### Utility of the TDF and COM-B model

Despite these limitations, the COM-B model and TDF offered a systematic, theory-driven approach to identify barriers and enablers to sexual health service use among university students. Although the COM-B and TDF helped to identify barriers and enablers at multiple conceptual levels, the BCW lacked clear guidance for teasing out how the contextual mechanisms function across different organizational settings. Although the COM-B and TDF helped to identify barriers and enablers at multiple conceptual levels, the two models lacked guidance for examining how the contextual mechanisms function across different organizational settings. Other researchers have had similar experiences in using the TDF to examine multi-level behavioural problems [54–58]. The TDF is a comprehensive framework for examining multi-level barriers and enablers but it is sometimes used with other frameworks to provide a more fully-defined understanding of multi-level determinants [54]. Future health services research using the TDF and COM-B Model may benefit from including an organizational-level framework to examine the contextual factors influencing individual behaviour.

Overall, by using the COM-B model of behaviour in combination with the TDF, we were able to first conceptualize the findings more broadly within students’ capability, opportunity, and motivation, and then use the TDF domains to provide a more granular understanding of the barriers and enablers. Using this deductive analysis approach can potentially restrict findings to the COM-B components and TDF domains; however, by combining the deductive analysis with an inductive thematic analysis, we were able to identify overarching themes of barriers and enablers to sexual health service use. The next step in this intervention design process is to use the BCW to select intervention components aimed at overcoming the barriers and enhancing the enablers identified in this study.

## Conclusion

Our findings highlight a range of factors related to students’ capability, opportunity and motivation that require attention to improve their use of sexual health services. It is clear that tailored, multi-level interventions are needed to target barriers and enablers at the individual, interpersonal and health system levels. Using a theory-based approach, we identified ten barriers and enablers to sexual health service use among university students related to students’ capability, opportunity and motivation for accessing these services. Based on these findings, we recommend that researchers, health care providers, and university administrators tailor sexual health service interventions to target the identified barriers and enablers to improve students’ use of sexual health services, and ultimately their overall health and well-being.

## Additional file

Additional file 1: Focus group and interview guides. (DOCX 20 kb)

## Abbreviations

BCW: Behaviour change wheel
CO: Beliefs about consequences
COM-B: Capability, opportunity, motivation and behaviour
E: Environmental context and resources
EM: Emotion
FG: Focus group
HIV: Human immunodeficiency virus
K: Knowledge
LGBTQ: Lesbian, gay, bisexual, transgender, queer
MAD: Memory, attention, and decision-making processes
OP: Optimism
Pap: Papanicolaou
RA: Research assistant
SI: Social Influences
STI: Sexually transmitted infection
TDF: Theoretical domains frameworks
