# Supplementary material for: Barriers and enablers to sexual health service use among university students: a qualitative descriptive study using the Theoretical Domains Framework and COM-B model
Source: BMC Health Serv Res. 2018 Jul 24;18:581. doi: 10.1186/s12913-018-3379-0 (PMC6057095; doi:10.1186/s12913-018-3379-0)
Supplement: Supplementary file 1 — Focus group and interview guides. (DOCX 20 kb) [file 12913_2018_3379_MOESM1_ESM.docx]

**Additional File 1 – Focus Group and Interview Guides**

**University Undergraduate Student Focus Group Guide**

1. **Knowledge**
2. Tell me about the sexual health services that are offered at your university? What do you know about these services? (Prompt- have you used the services?; what services exist, how do you make an appointment, what is the process for service use?)
3. How do you find out information about sexual health services offered at your university?
4. Do you or your friends use other sexual health services (not at your university)?

1. **Skill**
2. In your opinion, what knowledge or resources do you need to access sexual health services at your university? (i.e. communicating with receptionist, finding the information online, etc.)
3. Do you feel you and other undergraduate students have the knowledge or resources to access sexual health services at your university?

(prompt –are there any other skills that you need?)

1. **Social/Professional Role**
2. Do you feel like you have a responsibility to access sexual health services? Tell me about this. (prompt- Is there a responsibility to yourself? To others?)

1. **Beliefs about capabilities**
2. How easy or difficult is it to access sexual health services at your university? (prompt – what would make it easy or difficult for you?)
3. How confident do you feel in your ability to access sexual health services at your university?
4. What problems have you encountered (or do you foresee encountering) in accessing sexual health services?
5. **Beliefs about consequences**
6. How useful do you find the sexual health services offered at your university? (prompts- what are the benefits of university services?; Has your view on sexual health services changed over time?)
7. Are there any harms that can occur from using the sexual health services at your university? Are there any harms that can occur from NOT using the sexual health services at your university?
8. **Optimism**
9. How optimistic are you that students will use university sexual health services?
10. **Reinforcement**
11. Are there any incentives for you to access sexual health services at your university? What are they?
12. **Intentions**
13. On a scale of 1 to 10 and 10 being very important, how important do you think it is for you to access sexual health services at your university or in another setting (e.g. primary health care clinic)? Why?
14. **Goals**
15. Is accessing sexual health services at your university part of your health and wellness goals? (Prompt – why or why not?)
16. **Memory, attention and decision process**
17. Can you anticipate forgetting (or do you forget) that sexual health services are offered at your university? When do (would) you forget?
18. What would help make it easy to remember to use the sexual health services offered at your university?
19. What influences or triggers (what would influence or trigger) you to use the sexual health services at your university?
20. **Environmental context and resources**
21. What factors in the campus environment/student life would influence your decision or ability to access the sexual health services at your university? (location? Confidentiality/privacy?
22. What factors outside of the campus environment/student life influence your decision or ability to access the sexual health services at your university?
23. Are there competing tasks or time constraints that would influence your ability to access the sexual health services at your university?
24. **Social influences**
25. Do you ever discuss access sexual healthcare services with your family, friends, or sexual partners? (prompt-does their support influence your decision to access services?)
26. Would your family, friends, or sexual partners influence your decision to access sexual health services? How would they influence your decision? To what extent?
27. **Emotion**
28. Does discussing sexual health services ever evoke an emotional response in you? (prompt – would you feel worried or concerned about accessing sexual health services at your university?)
29. Thinking about yourself and how you normally feel as an undergraduate student to what extent do you feel motivated to access sexual health services? To what extent to you feel nervous to access sexual health services?
30. Would your family, friends, or sexual partners’ emotions ever affect your decision to access sexual health services at your university?
31. **Behavioural regulation**
32. Is discussing sexual health care matters something you do regularly in your daily life? (something you feel comfortable with)
33. What do you think is needed to ensure that you consistently access sexual health services at your university? (prompt –things specific to you, your university health centre, the university administration)

**Key Informant Interview Guide**

| **CAPABILITY** | |
| --- | --- |
| ***Psychological Capability*** | |
| *Knowledge* | Are you familiar with any guidelines or policies that university students and sexual health services? Can you describe what the guidelines or policies say? |
|  | PROMPT: Do you use any guidelines or policies to try to improve university students’ use of sexual health services? |
| *Behavioural Regulation* | Is discussing sexual health care matters something you do automatically in your practice or profession? |
|  | What do you think is needed to ensure that you consistently provide effective sexual health services to students? (prompt –things specific to you, your health centre, the administration) |
| *Memory, Attention, and Decision Process* | Are there situations when you think it would be difficult to provide sexual health services to students? (prompt – can you tell me what it is about these situations that make it difficult) |
|  | What influences or triggers (what would influence or trigger) you to provide sexual health care to students or advance the sexual health service policies? |
| ***Physical Capability*** | |
| *Skills* | What skills are needed to provide effective sexual healthcare to university students? Or what skills are needed to improve sexual healthcare to university students? |
|  | Do you feel you have the skills to provide effective sexual healthcare to university students? Do you feel you have the skills to advance sexual healthcare policy for university students?  (prompt –are there any other skills that you need?) |
| **OPPORTUNITY** | |
| ***Social Opportunity*** | |
| *Social influences* | Do you ever discuss sexual health services or policies with other physicians, nurses, or administrators in your clinic? |
|  | Would other clinicians in your clinic influence your decision to provide sexual healthcare services to students? How would they influence your practice? To what extent? |
|  | PROMPT: Do your colleagues value providing effective sexual healthcare services to students or improving sexual health service policy? |
| ***Physical Opportunity*** | |
| *Environmental Context and Resources* | What factors outside of your professional/practice environment would influence your ability to provide more effective sexual health care services or improve sexual health service policies? (Prompt – other departments in the university?) |
|  | Are there competing tasks or time constraints that would influence your ability to provide more effective sexual health care services or improve sexual health service policies? |
| **MOTIVATION** | |
| ***Automatic Motivation*** | |
| *Reinforcement* | Are there any incentives for you to provide sexual health services to students at your health centre? What are they? |
|  | When you provide sexual health care to students do you feel like you are making a difference? Why or why not? |
| *Emotion* | Does discussing sexual health services for university students ever evoke an emotional response in you? (prompt – would you feel worried or concerned about providing sexual health services?) |
|  | Thinking about yourself and how you normally feel as a professional that works with university students, to what extent do you feel inspired to provide sexual health services or advance policy? To what extent to you feel nervous to provide sexual health services or advancing policy? |
| **Reflective Motivation** | |
| *Social/Professional Role And Identity* | What responsibilities do you have as a health care provider or university administrator to provide sexual healthcare services? |
|  | How is sexual healthcare provision and/or policy development consistent or inconsistent with your profession? |
|  | How compatible is the provision of sexual healthcare with your profession? |
| *Beliefs About Capabilities* | How confident do you feel in your ability to provide sexual health services to university students? |
|  | How easy or difficult is it to provide sexual healthcare services to university students or focus on advancing sexual health service policy for university students? How easy or difficult is it to improve provide sexual healthcare services to university students? (prompt – what would make it easy or difficult for you?) |
| *Beliefs about Consequences* | Do you find the university health clinics’ sexual health services useful? |
|  | In the socio-political context of your clinic, is there sufficient financial support to provide sexual health services? |
|  | *What do you think are the benefits of these types of services? (prompts – Are there any particular patient benefits, financial benefits, HCP benefits or administration benefits? Is there enough time to provide effective sexual health care?)* |
|  | *Are there any harms that can occur from providing sexual health services to university students? (prompts – is there any potential harm for the patient, health care professional or the campus health clinic?)* |
| *Optimism* | How optimistic are you about the future of sexual health services at university health centres? |
| *Intentions* | On a scale of 1 to 10 and 10 being very important, how important do you think it is for you to provide sexual healthcare services to university students at your health clinic? Why? |
| *Goals* | Would the goal of improving sexual health services be compatible with your usual practice? (Prompt - why?) |
|  | Generally, how often does covering something else on your agenda take precedence of sexual health service provision or policy development? (What usually takes precedence?) |
